# Supplementary material for: L1 and L2 reading skills in Dutch adolescents with a familial risk of dyslexia
Source: PeerJ. 2017 Oct 16;5:e3895. doi: 10.7717/peerj.3895 (PMC5647862; doi:10.7717/peerj.3895)
Supplement: Supplemental Information 1 [file peerj-05-3895-s002.docx]

**Appendix A**

*English Spelling test*

1. An apple a day keeps the doctor away.
2. He plays the guitar very well.
3. He was born in Germany.
4. After a long day I am very tired.
5. The teacher writes on a blackboard.
6. Every day they build on the new bridge.
7. These children are very young.
8. Where do you live?
9. I have been to six different countries.
10. You can only eat typically English dishes in a pub.
11. I think that we're done.
12. Every Thursday I visit my grandparents.
13. Jane likes to climb mountains.
14. The spaghetti is delicious.
15. Vegetables are very healthy.
16. Have you learnt any new words today?
17. I have eaten enough candy for today.
18. I love eating ice cream.
19. Last week Alice and John got married in a church.
20. Sally is going to buy a house in London.
