# Supplementary material for: L1 and L2 reading skills in Dutch adolescents with a familial risk of dyslexia
Source: PeerJ. 2017 Oct 16;5:e3895. doi: 10.7717/peerj.3895 (PMC5647862; doi:10.7717/peerj.3895)
Supplement: Supplemental Information 2 [file peerj-05-3895-s003.docx]

**Appendix B**

| Table B1  *Descriptive Statistics of the Unstandardized Data.* | | | | | | | | | | |
| --- | --- | --- | --- | --- | --- | --- | --- | --- | --- | --- |
| Measure | All Participants  (*N =* 81) | | LRnonDys  (*n*  = 27) | | LRDys  (*n*  = 4) | | HRnonDys  (*n*  = 25) | | HRDys  (*n* = 25) | |
|  | *M (Sd)* | Min – Max | *m (sd)* | min - max | *m (sd)* | min - max | *m (sd)* | min - max | *m (sd)* | min - max |
| Dutch Words RF  Items correct / 1 min.  (max = 145) | 76.65 *(17.35)* | 22 - 110 | 87.11 *(9.84)* | 70 - 103 | 66.75 *(16.21)* | 47 - 84 | 84.32 *(12.10)* | 58 - 110 | 59.28 *(14.30)* | 22 - 80 |
| Pseudoword RF  Items correct / 2 min.  (max = 145) | 70.98 *(24.45)* | 10 - 124 | 90.41 *(17.23)* | 57 - 124 | 49.75 *(12.71)* | 39 - 68 | 77.00 *(17.91)* | 31 -110 | 47.36 *(14.55)* | 10 - 69 |
| Loanword RF  Items correct / 1 min.  (max = 116) | 65.47 *(18.05)* | 3 - 101 | 76.19 *(11.19)* | 49 - 100 | 55.00 *(10.39)* | 40 - 64 | 70.76 *(11.05)* | 50 - 101 | 50.28 *(19.94)* | 3 - 85 |
| English Word RF  Items correct / 1 min.  (max = 108) | 63.85 *(15.08)* | 17 - 97 | 73.00 *(10.69)* | 51 - 92 | 55.75 *(12.87)* | 48 - 75 | 67.48 *(10.23)* | 48 - 97 | 51.64 *(15.33)* | 17 - 82 |
| Dutch Spelling  Accuracy (max = 90) | 73.10 *(12.45)* | 38 - 89 | 80.70 *(5.44)* | 67 - 88 | 65.75 *(4.19)* | 60 - 70 | 77.44 *(7.24)* | 62 - 89 | 61.72 *(14.19)* | 38 - 83 |
| English Spelling  Accuracy (max = 20) | 11.88 *(5.26)* | 1 - 20 | 15.59 *(3.31)* | 6 - 20 | 7.75 *(2.99)* | 4 - 11 | 13.88 *(3.22)* | 4 - 18 | 6.52 *(4.06)* | 1 - 15 |
| Dutch Vocabulary  Accuracy (max = 70) | 49.60 *(7.34)* | 18 - 62 | 52.15 *(8.98)* | 18 - 62 | 47.50 *(5.92)* | 41 - 53 | 50.24 *(5.40)* | 41 - 62 | 46.56 *(6.40)* | 33 -55 |
| English Vocabulary  Accuracy (max = 228) | 135.98 *(26.53)* | 63 - 186 | 144.67 *(24.97)* | 88 - 186 | 121.50 *(20.14)* | 103 - 150 | 140.00 *(21.62)* | 90 - 175 | 124.88 *(29.80)* | 63 - 181 |
| Spoonerisms  Accuracy (max = 20) | 17.32 *(3.19)* | 4 - 20 | 19.19 *(0.92)* | 17 - 20 | 17.25 *(3.59)* | 12 -20 | 18.08 *(2.20)* | 13 - 20 | 14.56 *(3.75)* | 4 - 20 |
| Spoonerisms  RT (Log-transformed) | 3.69  *(0.34)* | 3.06 - 4.67 | 3.39  *(0.18)* | 3.06 - 3.83 | 3.97  *(0.28)* | 3.61 - 4.29 | 3.65 *(0.24)* | 3.09 - 3.94 | 4.01 *(0.25)* | 3.59 - 4.67 |
| RAN  Items correct / 1 min. | 145.28 *(26.08)* | 98.00 - 230.77 | 155.68 *(27.10)* | 111.11 - 214.29 | 115.87 *(8.23)* | 107.14 - 125.22 | 150.15 *(27.20)* | 103.45 - 230.77 | 133.87 *(17.24)* | 98.00 - 166.67 |
| Digit Span - Forward  Accuracy (max = 16) | 8.56 *(1.89)* | 5 - 13 | 9.74 *(2.07)* | 6 - 13 | 8.00 *(1.41)* | 6 - 9 | 8.36 *(1.52)* | 5 - 11 | 7.56 *(1.42)* | 5 - 10 |
| Digit Span - Backward  Accuracy (max = 14) | 5.63 *(1.91)* | 2 - 11 | 6.48 *(1.87)* | 3 - 10 | 4.25 *(0.50)* | 4 - 5 | 5.80 *(2.08)* | 2 - 11 | 4.76 *(1.42)* | 2 - 7 |
| *Note.* Missing data were imputed with the expectation maximization method for Dutch Spelling in 1 case, RAN in 2 cases and Spoonerisms RT in 4 cases. LRnonDys = Low Risk without Dyslexia, LRDys = Low Risk with Dyslexia, HRDys = High Risk with Dyslexia, HRnonDys = High Risk without Dyslexia, RF = Reading Fluency, RT = Reaction Time, RAN = Rapid Automatized Naming. | | | | | | | | | | |
